# Statistical learning of target location guides attention proactively

**DOI:** 10.3758/s13423-025-02710-9

**Published:** 2025-05-21

**Authors:** Aidai Golan, Aniruddha Ramgir, Dominique Lamy

**Affiliations:** 1 School of Psychological Sciences, Tel Aviv University, Tel Aviv-Yafo, Israel; 2 Sagol School of Neuroscience, Tel Aviv University, Tel Aviv-Yafo, Israel; 3 Department of Psychology, Tel Aviv University, Ramat Aviv, POB 39040, 69978 Tel Aviv, Israel

**Keywords:** Statistical learning, Proactive attentional guidance, Task context, Selection history, Visual search

## Abstract

Our perceptual system is highly sensitive to statistical regularities in our environment. In particular, we respond faster to targets that appear in frequently attended locations—a phenomenon known as target-location probability learning (LPL). Is attention proactively aligned with the high-probability target location, or reactively allocated to that location when the learning context is detected? The studies that addressed this question tested whether a spatial attentional bias learned in one task transfers to another. However, they yielded conflicting findings and were open to alternative accounts. We reexamined whether LPL-guided attention is proactively allocated to the high-probability target location while addressing these previous studies’ potential caveats, in two experiments. During learning, the search target appeared more often at one location than elsewhere, and during extinction, all search target locations were equiprobable. In both learning and extinction, letter-probe trials were interspersed among the search trials. We found that LPL acquired during search transferred to the letter-probe task during both learning and extinction. Importantly, during extinction, participants continued to prioritize the previously high-probability location on both search and letter-probe trials, even when they were informed after the learning phase that the bias would be discontinued and were asked to start their search at the location indicated by an arrow precue. We conclude that LPL guides attention proactively and inflexibly.

**Supplementary Information:**

The online version contains supplementary material available at 10.3758/s13423-025-02710-9.

How we deploy attention depends on our current goals, on stimulus salience, and on what we attended to in the past—that is, on our selection history. Current visual-attention theories posit that all three mechanisms combine to determine the overall activation of each location on a priority map. According to these integrated-priority-map theories (e.g., Awh et al., 2012; Luck et al., 2021; Wolfe, 2020; see Lamy et al., in press, for review), the most strongly activated location receives attention in a winner-take-all fashion.

A selection-history phenomenon that has recently received much interest is location-probability learning (henceforth, LPL, e.g., Geng & Behrmann, 2002, 2005; Jiang, 2018): People respond faster to targets that appear in frequently attended locations. According to integrated-priority-map theories, this occurs because the attentional weight of the high-probability location on the priority map becomes larger as learning develops. Thus, these theories assume that after learning is established, attentional enhancement at the high-probability location is proactive: It should benefit any object at that location (not just the target), irrespective of context. Recently, Jiang (2018) suggested that instead, repeatedly attending to a location creates a search habit, such that “every time a target is successfully detected, the most recent shifts of attention that lead to target detection are reinforced, increasing the likelihood that such vectors of attentional shift would occur again” (p. 116). Importantly, Jiang (argued that like habits, LPL transfers only to tasks that require similar behaviors. Accordingly, she showed that LPL established in a search task generalizes to a different search task but not to a foraging task (e.g., Jiang et al., 2015). 

Addleman et al., (2018, 2019) reexamined this conclusion. They noted that transfer of learning may have failed in Jiang et al.’s (2015) study because participants were trained on one task, and generalization was tested on a subsequent task. This two-phase design entailed that early into the test phase, participants knew their task had changed and could therefore adjust their behavior accordingly. To determine whether transfer of learning occurs when participants cannot anticipate the next task, Addleman et al., (2019, Experiment 1) intermixed a letter-search task where the bias was present and an image-search task where the bias was absent. On letter-search trials, participants searched for a *T* among *L*s. On image-search trials, they searched for a target (e.g., a forest) among four images, each in a different quadrant and presented for 217 ms. Then, these images were replaced with four arrows and participants reported the orientation of the arrow that replaced the target image. The experiment included two phases: learning and extinction. During learning, the target was more likely to appear in one (high-probability) quadrant than in the other (low-probability) quadrants on letter-search trials, whereas the target on image-search trials was equally likely to appear in each quadrant. During extinction, all target locations were equiprobable for both tasks. 

Addleman et al. (2019) reasoned that if LPL is proactive, the bias learnt in the letter-search task should generalize to the image-search task: Responses to the arrow should be fastest for target images at the high-probability location of the *T* target. Letter-search performance confirmed that participants learned the probability imbalance and that the acquired bias was long-lasting. However, this bias did not manifest in the image-search task during either phase. The authors concluded that LPL does not induce anticipatory shifts of attention.

Huang et al., (2022, Experiment 1) used a similar rationale yet arrived at the opposite conclusion. Participants performed a search task (2/3 of the trials) and a probe task (1/3), randomly intermixed. On search trials, participants searched for a unique shape that was presented more often at one location. On probe trials, each location contained a shape enclosing a dot and participants had to respond whenever one of the dots was offset. The critical offset, when present, was equally likely to occur at each location. The authors found that responses to the dot offset were faster at the high- versus low-probability target locations and concluded that LPL guides attention proactively.

The two studies led to opposite conclusions, but their findings are open to alternative explanations. In Addleman et al.’s (2019) image-search task, participants searched for a highly discriminable image, and the response-relevant information appeared only after the image display was replaced with an arrow display (after 217 ms). As the target image was defined by its gist (a forest), detecting it probably did not require attention. Thus, participants might have had enough time to detect the target image and disengage from the high-probability quadrant within the 217 ms that preceded the arrow-display onset, explaining why no LPL was detected. However, the results of the second experiment, where the task roles were reversed and the probability manipulation pertained to the image-search task, may mitigate this alternative account. On the one hand, the bias was observed in the image-search task during learning, suggesting that 217 ms may not suffice to fully disengage attention from the high-probability location. On the other hand, the bias was found only during learning and not during extinction. A reasonable possibility is that disengaging attention from the scene at the high-probability location may have been slow in the learning phase of Experiment 2 because that location most often contained the target, unlike in the extinction phases of Experiments 1 and 2.

In Huang et al.’s (2022) study, there was no extinction phase: As target location was more likely to repeat on consecutive trials in the high- versus low-probability locations, the results may reflect intertrial priming (e.g., Talcott et al., 2022; Toledano & Lamy, 2025), rather than LPL. Huang et al. (2022) addressed this issue by never presenting the critical probe at the high-probability location on successive trials. However, this procedure does not suffice to eliminate intertrial priming, whose influence typically lasts over 5–8 trials (Maljkovic & Nakayama, 1996; see Golan & Lamy, 2023, for long-lasting intertrial priming in LPL).

Here, we investigated whether LPL guides attention proactively while addressing the potential caveats of these previous studies. Unlike Addleman et al. (2019), we measured participants’ attention early, before it could be shifted to a different location. Unlike Huang et al. (2022), we included both a learning and an extinction phase to control for intertrial priming. In addition, the task used to test transfer of learning (a probe-letter report task, henceforth “probe task”) was highly dissimilar from the task in which the bias was learnt (a search task).

On search trials, participants looked for a shape-defined target and reported the location of a dot inside it. The search target was presented more often at one (high-probability) location (60%) than at the other (low-probability) locations (10% each) during learning and appeared equally often at each location during extinction. During both phases, on probe trials, a letter, briefly presented (100 ms) and then masked, replaced each shape and participants reported the letters they remembered (Gaspelin et al., 2015). Search trials (2/3) and probe trials (1/3) were randomly intermixed.

We expected to observe LPL on search trials in both the learning and extinction phases (replicating Addleman et al., 2019). Crucially, if LPL is proactive, more letters should be reported from the high- versus low-probability locations on probe trials during both phases. Finding no such advantage on probe trials would indicate that participants deploy their attention to the high-probability location reactively, in response to the display that characterizes the task on which learning was established.

## Experiment 1

### Methods

#### Sample-size selection

Addleman et al. (2019) used a sample of 32 participants, which should suffice to detect an effect size of *d* = 0.45, with an alpha of 0.05 and power of 0.8. These authors found an effect size of *d* = 1.3 on search trials that did not transfer to the probe task. As our rationale was that their null finding resulted from the fact that participants may have had time to disengage from the high-probability location, it was reasonable to aim for an effect size of *d* = 0.45 when such disengagement was prevented, and we therefore recruited 32 participants.

#### Participants

The participants were 32 students (23 women, mean age = 23 years, *SD* = 2.9) who participated in the experiment for course credit. All but four participants were right-handed and all reported normal or corrected-to-normal visual acuity. The research was approved by University Ethics Committee, 0000285–5 and complies with all national and international (e.g., Declaration of Helsinki) ethical regulations. Informed consent was obtained from all participants.

#### Apparatus

The experiment took place in a dimly lit room. Stimuli were presented on 23-in. screen, using 1,920 × 1,080 resolution and 120-Hz refresh rate. Responses were collected via the computer keyboard and mouse. Participants were seated at approximately 60 cm from the monitor.

#### Stimuli

##### Search task

**Fig. 1:**
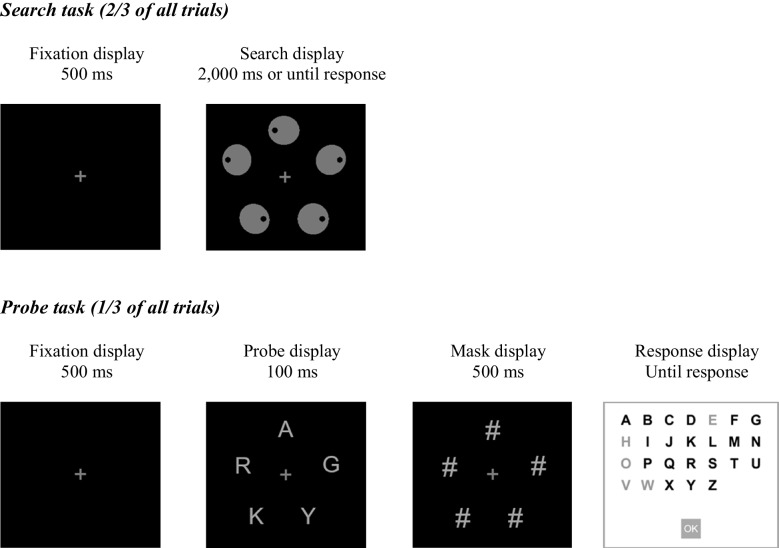
Sample displays in the search and probe tasks in Experiment 1. On search task (upper panel), participants searched for the perfect circle and responded to the position (right or left) of the dot inside it. On probe task (lower panel), participants reported the letters they remembered from the probe display. Not drawn to scale

Each search display (Fig. 1, upper panel) contained a central cross surrounded by five shapes, placed equidistantly on an imaginary circle (2.4° in radius): a circle (1.4° in diameter), which was the target, and four ovals. Two of these ovals subtended 1.4° × 1.29° of visual angle and were rotated by 45° from the vertical, one to the right and the other to the left; the remaining two ovals subtended 1.4° × 1.27°,^1^ with the larger axis either horizontally or vertically oriented. Each shape contained a black dot (0.4° in diameter), 0.45° away of the shape center on either the left or the right. All items were gray (RGB: 128, 128, 128) against a black background.

##### Probe task>

Each probe display consisted of five letters from the English alphabet (1.15° in height), at the same locations as the search-display shapes (see Fig. 1, lower panel). The mask display was similar but contained hash signs (1.15° in height) instead of letters. The response display contained the full English alphabet and an “OK” button (all 1.73° in height).

#### Procedure

##### Search task

Each search trial began with the fixation cross (500 ms), followed by the search display until response or 2,000 ms. A new trial began after a 500-ms blank inter-trial interval. Participants reported whether the target circle contained a left or right dot by pressing the “1” or “2” keys, respectively, as fast and as accurately as possible. An incorrect response was followed by a beep (225 Hz). Eye movements were not monitored, but participants were explicitly instructed to maintain fixation at the plus sign.

##### Probe task

Each probe trial began with the fixation cross (500 ms), followed by the brief probe display (100 ms), the mask display (500 ms), and finally the response display. Participants were instructed to use the mouse to select only letters they saw without guessing and were informed that they could deselect a letter by clicking on it again. Each selected letter turned from black to gray. Participants clicked on the “OK” button to confirm their selection on the response display.

#### Design

The experiment included three phases: practice, learning, and extinction (in this order). The practice phase consisted of two blocks: the first one included 20 search trials, and the second one included 36 trials, 24 search trials, and 12 probe trials. In both practice blocks, the target was equally likely to occur at each location on search trials.

In both the learning and extinction phases, 2/3 of the trials were search trials and 1/3 were probe trials. During the learning phase (360 trials divided into three blocks), on search trials, the target was presented in one location more often (60% of the trials, high-probability location) than in the others (10% of the trials each, low-probability locations). The high-probability location remained the same for each participant throughout the learning phase and was counterbalanced across participants. During the extinction phase (360 trials divided into three blocks), all target locations were equiprobable. The dot position in all search trials was randomly selected on each trial. Participants were allowed a short self-paced break of up to 3 min after each block of trials.

#### Index of the relative priorities on probe trials

Our index of the relative priorities accruing to the different locations in the probe display was the proportion of probes reported from the high- versus low-probability locations out of the total number of correctly reported probes. With this method, the sum of the proportions of the probes reported from each location always adds up to 100% (unlike with the method used by Talcott et al., 2022). Our method therefore allows one to compare different conditions (e.g., arrow-cue vs. neutral-cue conditions in Experiment 2), where the average number of correctly reported letter might differ (see Wirth et al., 2023, for details).

### Results

**Fig. 2:**
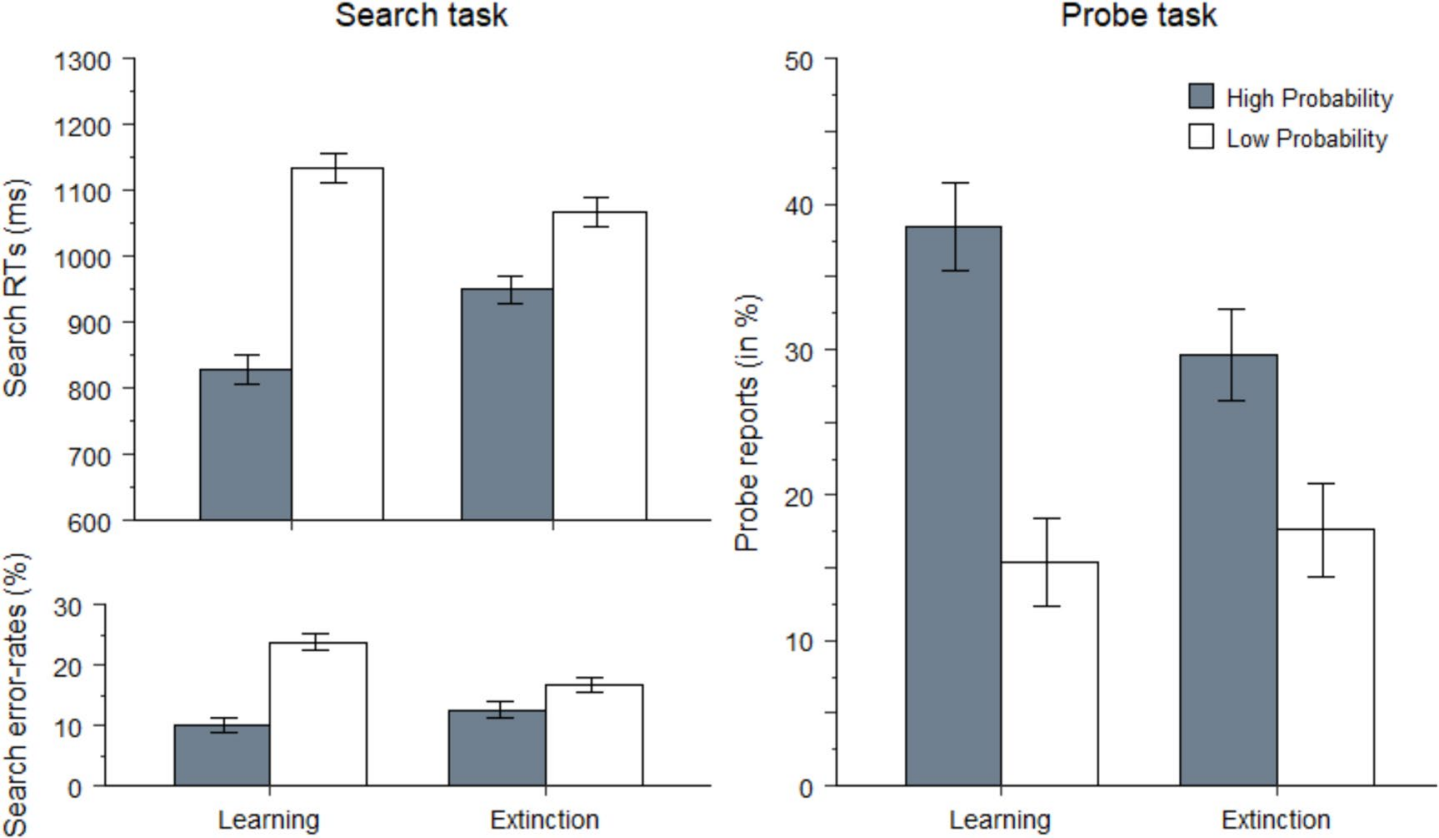
Left panels: Reaction times (in ms) and mean error rates (in percentages) on search trials as a function of target-location probability (high vs. low). Right panel: Probe reports from the target location (in percentages) on probe trials (right panel) as a function of reported-probe location (high vs. low probability) during the learning and extinction phases of Experiment 1

We conducted a paired-sample *t* test (high- versus low-probability target location) on mean RTs, accuracy, and probe reports (Fig. 2), separately for each phase.^2^

#### Learning phase

For search-RT analyses, error (15%) and RT-outlier trials (2.2%) were excluded. As expected, search was faster for high- versus low-probability-locations targets, 828 ms versus 1,134 ms, *t*(31) = 7.8, *p* < 0.001, *d* = 1.4, and more accurate, 90% versus 76%, *t*(31) = 6.5, *p* < 0.001,* d* = 1.1. On *probe* trials, participants correctly reported an average of 1.4 letters per trial (*SD* = 0.5) and reported more letters from the high- versus low-probability locations, 38% versus 15%, *t*(31) = 4.2, *p* < 0.001, *d* = 0.7.

#### Extinction phase

For search-RT analyses, error trials (16%) and RT outliers (1.1%) were excluded. The bias survived in the extinction phase: Search was faster for high- versus low-probability-location targets, 949 ms versus 1,067 ms, *t*(31) = 3.2, *p* = 0.002, *d* = 0.6, and tended to be more accurate, 87% versus 83%, *t*(31) = 1.9, *p* = 0.06, *d* = 0.3. On probe trials, participants correctly reported an average of 1.5 letters per trial (*SD* = 0.7). Crucially, LPL was also evident on these trials, with more letters reported from the high- versus low-probability locations, 30% versus 18%, *t*(31) = 2.2, *p* = 0.03, *d* = 0.4.

The findings are straightforward: LPL transferred from a search task to an entirely different letter-report task—in both learning and extinction, precluding the possibility that intertrial priming rather than LPL explained our findings. These results indicate that attention is proactively guided towards high-probability-target locations.

## Experiment 2

In Experiment 1, we used a strong probability manipulation (60% vs. 10%). Therefore, participants may have noticed the imbalance. If so, since most trials were search trials, participants may have strategically aligned their attention with the high-probability location before the upcoming display onset, on both search and probe trials.

Recent findings from Golan and Lamy (2023, Experiment 4) do not support this possibility: The authors showed that LPL survived extinction even when participants were explicitly discouraged to attend to the high-probability location. However, the probability manipulation in that study was less conspicuous (the probability imbalance was 50% vs. 12.5% and concerned search-display quadrants, rather than individual-item locations). Thus, the fact that strategic allocation of attention to the high-probability region did not account for LPL in that study does not ensure that this was also true here.

We tested this alternative account in Experiment 2. It was similar to Experiment 1 except that after the learning phase, we disclosed the probability manipulation and informed participants that it would be discontinued in the next phase. A cue, either a central arrow pointing at one item location (arrow-cue trials, 50%) or a central square (neutral-cue trials, 50%), appeared before each display. Participants were told that they should inspect the cued location first on arrow-cue trials, and any location they chose on neutral-cue trials. The arrow cue was not predictive of the target location, but participants were not given any information about predictiveness.

We expected to replicate the results of Experiment 1 during learning. Our main interest was in whether attention would be biased toward the (previously) high-probability location during extinction, despite our instructions. On search trials, we expected a high-probability location benefit on neutral and invalid-cue trials. On valid-cue trials, we expected a small effect, if any, because when the cue and target locations coincided, LPL was unlikely to facilitate search beyond the benefit of the valid cue. On probe trials, participants did not have to search, and the cue directed their attention to one of the locations. Therefore, we expected the letter at the cued location to be correctly reported on most trials. Crucially, we expected more uncued letters to be reported from the (previously) high- versus low-probability location—and likewise, on neutral-cue trials.

### Methods

#### Sample-size selection

We conducted a power analysis to determine the required sample size based on the critical effect of LPL on probe reports during extinction from Experiment 1 (using G*Power; Faul et al., 2007). Note that since Experiment 2 had half the number of probe trials in the extinction phase relative to Experiment 1, we bootstrapped 50% of the data from the latter experiment to estimate the size of the critical effect in Experiment 2. To do that, we randomly selected 50% of the data from Experiment 1, with replacement, 1,000 times, and calculated the *p* value and Cohen’s *d* for the critical effect in each iteration. The bootstrapping analysis yielded an average *p* value of 0.02 and an average Cohen’s *d* of 0.4 (compared with *p* = 0.03 and *d* = 0.4 for the full dataset from Experiment 1). Based on the bootstrapped effect size (Cohen’s *d* = 0.4), the power analysis indicated that a sample size of 42 participants would be required to detect this effect with 80% power and an alpha level of 0.05.

#### Participants

Participants were 42 university students (38 women, mean age = 24 years, *SD* = 3.3) who participated for course credit. All but one was right-handed and all reported normal or corrected-to-normal visual acuity. Informed consent was obtained from all human participants.

#### Apparatus, stimuli, procedure, and design

**Fig. 3:**
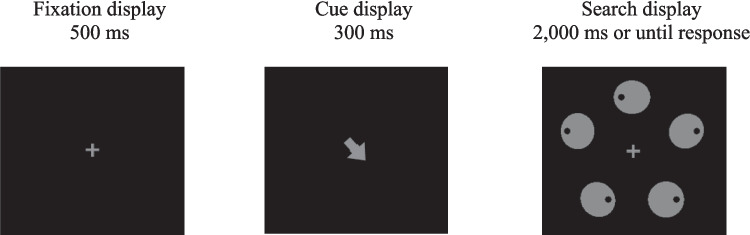
Sequence of events on a sample search trial during the extinction phase of Experiment 2. On half of the trials, the search or the probe display was preceded by the cue display. The example shows a search trial where the cue is valid (i.e., it points to the location of the target). Not drawn to scale

The apparatus, stimuli, procedure, and design were similar to those of Experiment 1, except for the following changes. A central arrow cue (1.4° long × 1.05° wide) was introduced on half of the trials of the extinction phase (see Fig. 3). When present, the cue was equally likely to point to any of the five locations of the upcoming display, irrespective of whether it was a search or a probe display. When the cue was absent, a filled square (0.45° in side) appeared at the center instead of an arrow cue.

After the learning phase was over, the experimenter entered the room, informed the participant that the target had been more likely to appear at one location than at the others during the previous phase and that it would be discontinued in the second part of the experiment. In addition, the experimenter instructed participants to first inspect the location indicated by the arrow. The arrow cue was not predictive of the target location, but participants were not given any information about its predictiveness.

#### Index of the relative priorities on probe trials

The index of the relative priorities accruing to the different locations on probe trials was calculated separately for neutral- and for arrow-cue trials, and for the latter, separately for trials in which the cued and high-probability locations coincided versus did not coincide. As in Experiment 1, we compared the proportion of reports from the high- versus average low-probability location out of the total number of correctly reported probes in each case, but when the cued and high-probability locations did not coincide, we compared the proportion of probes reported from the cued (low-probability), uncued high-probability and average uncued low-probability location.

### Results and discussion

Two participants were replaced: one because of a technical error and the other as an accuracy outlier (49% vs. *M* = 82%, *SD* = 8%). The data from three participants were excluded from the extinction search-trials analysis because one design cell was empty. These were not replaced because they were excluded after data collection was completed.

#### Learning phase

**Fig. 4:**
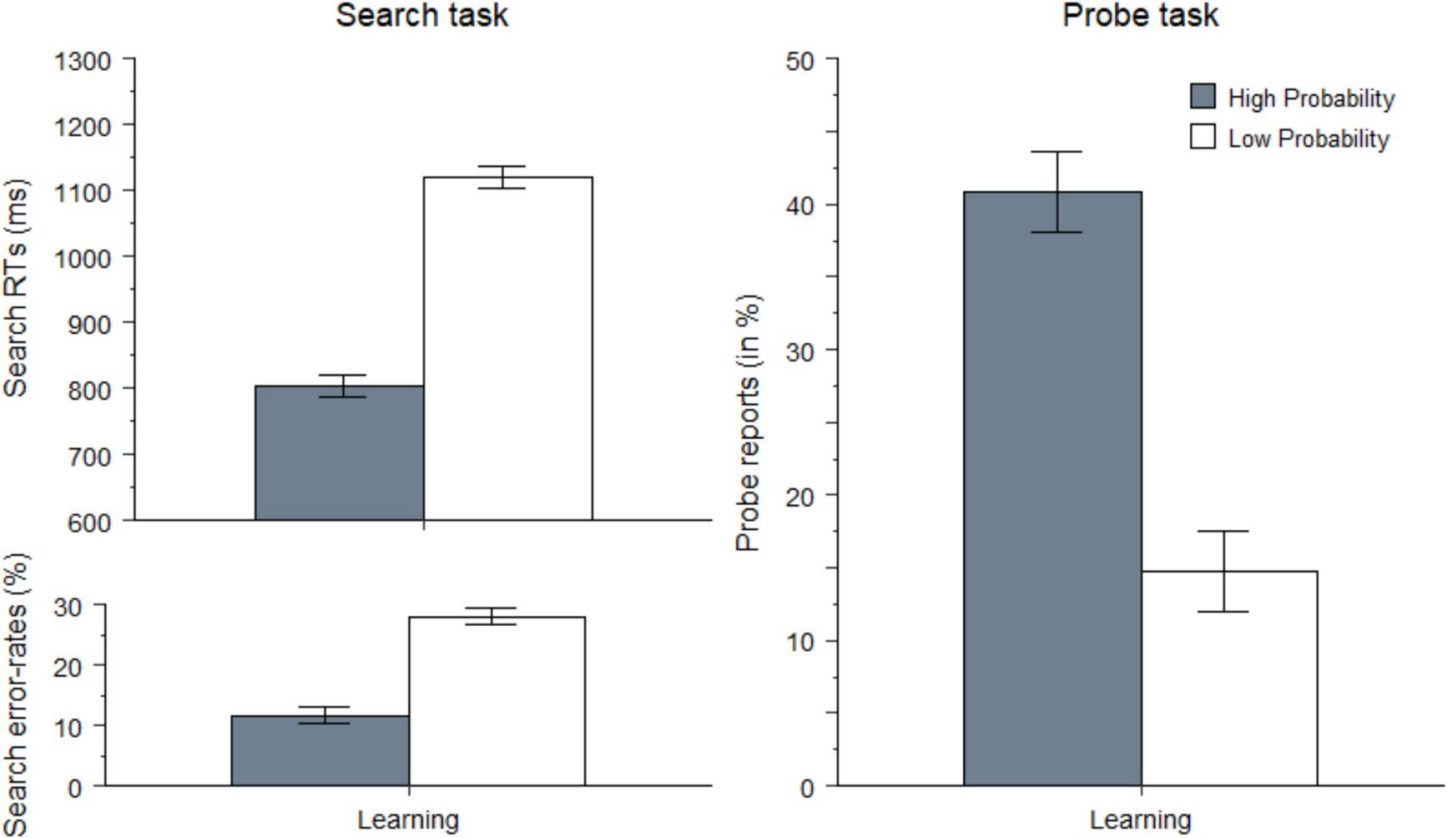
Left panels: Reaction times (in ms) and mean error rates (in percentages) on search trials as a function of target-location probability (high vs. low). Right panel: Probe reports (in percentages) on probe trials as a function of reported-probe location (high vs. low probability) during the learning phase of Experiment 2

The results of Experiment 1 were fully replicated (Fig. 4). For *search*-trial RT analyses, error (18%) and RT-outlier trials (2%) were excluded. Search was faster for high- versus low-probability-location targets, 803 ms versus 1,119 ms, *t*(41) = 13.2, *p* < 0.001, *d* = 2, and more accurate, 88% versus 72%, *t*(41) = 8.4, *p* < 0.001, *d* = 1.3. On *probe* trials, participants correctly reported an average of 1.4 letters per trial (*SD* = 0.4). Again, learning transferred to the probe task: Participants reported more letters from the high- versus low-probability locations, 41% versus 15%, *t*(41) = 6.6, *p* < 0.001, *d* = 1.

#### Extinction phase

##### **Search trials**

Error (23%) and RT-outlier trials (1%) were excluded from RT analyses. We conducted an analysis of variance (ANOVA), with target location (high vs. low probability) and cue condition (valid, invalid and neutral) as within-subject factors.

##### Reaction times

 The significant main effect of cue validity*, F*(2,76) = 143.6, *p* < 0.001, ${\eta }_{p}^{2}$= 0.8, confirmed that participants followed the cuing instruction: performance was faster on valid- versus neutral-cue trials, 823 ms versus 1,073 ms, *t*(38) = 12.6, *p* < 0.001, *d* = 2, and on neutral- versus invalid-cue trials, 1,073 ms versus 1,192 ms, *t*(38) = 8.7, *p* < 0.001, *d* = 1.4. Crucially, search was again faster for targets at the high- versus low-probability locations, *F*(1,38) = 19.5, *p* < 0.001, ${\upeta }_{p}^{2}$ = 0.3, and this effect interacted with cue validity, *F*(2,76) = 6.1, *p* = 0.003, ${\upeta }_{p}^{2}$ = 0.14. Follow-up analyses revealed that LPL did not reach significance on valid-cue trials, 35 ms, *t*(38) = 1.7, *p* = 0.09, *d* = 0.3, but was significant on both neutral-cue trials, 127 ms, *t*(38) = 4.9, *p* < 0.001, *d* = 0.8, and invalid-cue trials, 62 ms, *t*(38) = 2.8, *p* < 0.001,* d* = 0.4, with a significant difference between the latter two conditions, *F*(1,38) = 7.5, *p* = 0.009, ${\upeta }_{p}^{2}$= 0.16.

##### Accuracy

**Fig. 5:**
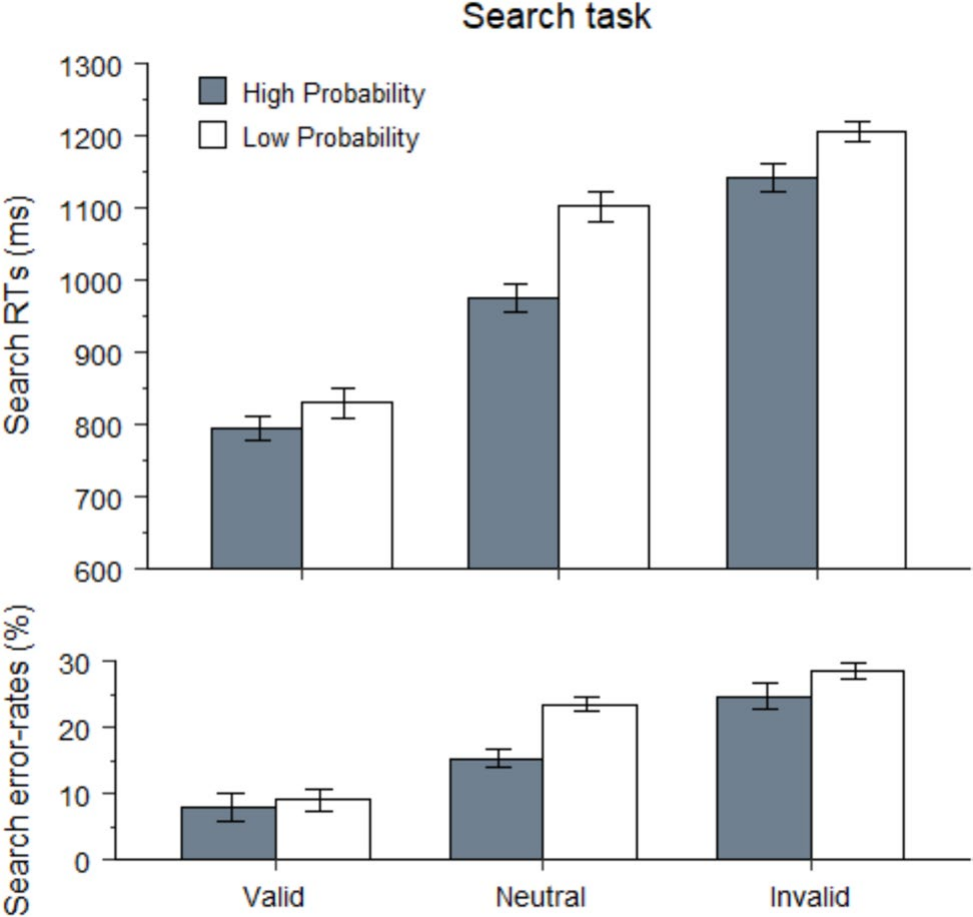
Reaction times (in ms) and mean error rates (in percentages) on search trials as a function of cue validity (valid, neutral, invalid) and target-location probability during the extinction phase of Experiment 2

 The accuracy data mirrored the RT data. The main effect of cue validity was significant, *F*(2,82) = 61, *p* < 0.001, ${\upeta }_{p}^{2}$= 0.6, with higher accuracy on valid- versus neutral-cue trials, 92%, versus 81%, *t*(41) = 8.4, *p* < 0.001, *d* = 1.3, and on neutral versus invalid-cue trials, 81% versus 73%, *t*(41) = 5.7, *p* < 0.001, *d* = 0.9. The main effect of target location was also significant, *F*(1,41) = 8.9, *p* = 0.005, ${\upeta }_{p}^{2}$= 0.18, with higher accuracy for high- versus low-probability-location targets, 84% versus 80%. The interaction between the two factors approached significance, *F*(2,82) = 3, *p* = 0.056, ${\upeta }_{p}^{2}$= 0.07, with numerical trends similar to the RT data (Fig. 5).

#### Probe trials

**Fig. 6:**
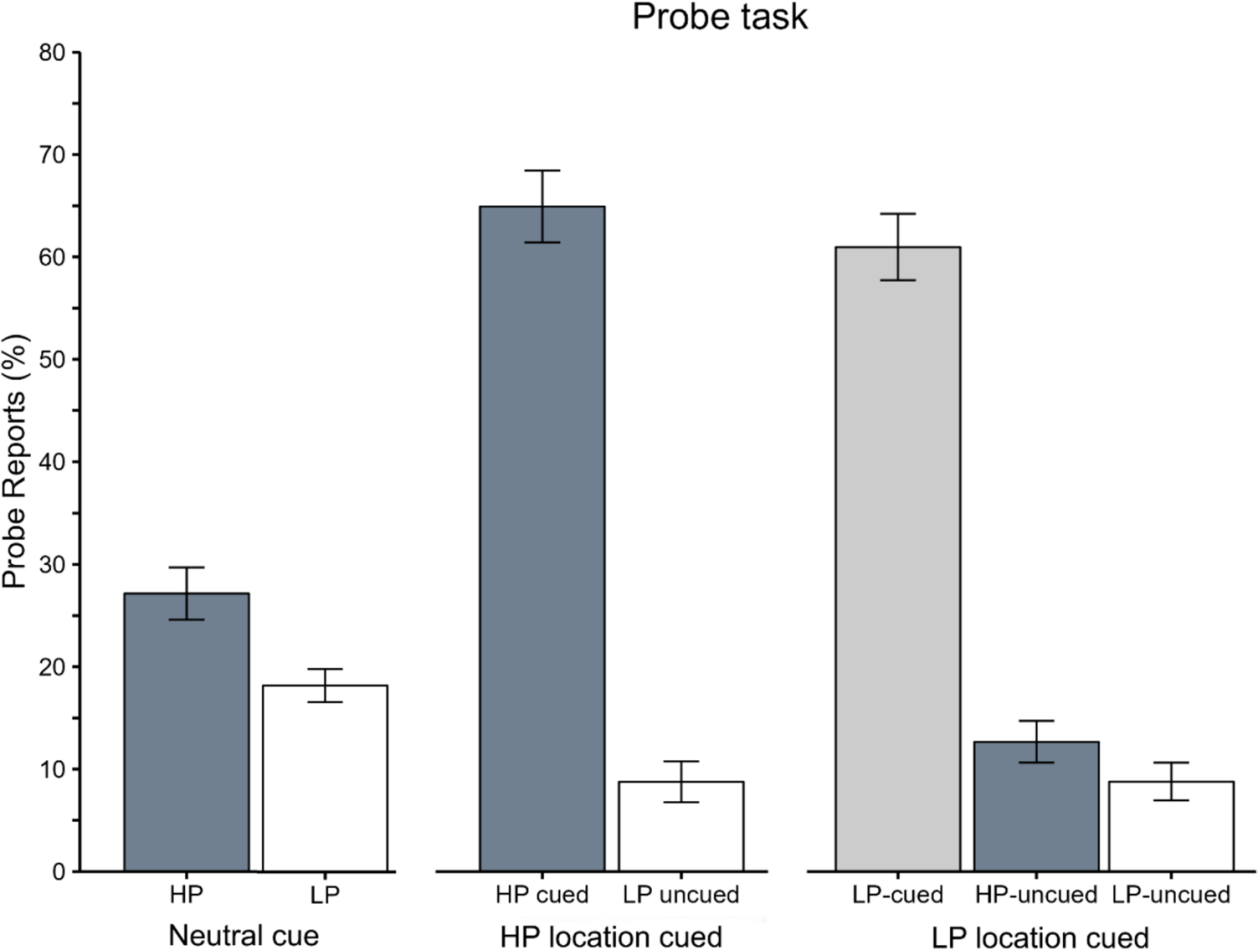
Probe reports (in percentages) from the high- versus low-probability locations as a function of cue type (neutral-cue vs. arrow-cue trials) on the probe trials of the extinction phase in Experiment 2. On arrow-cue trials, the cue coincided with either the high-probability location (middle bars − HP location cued) or a low-probability location (right bars − LP location cued)

Probe results are presented in Fig. 6. Since there was no target, there were no cue-validity conditions. Participants reported more letters on neutral-cue than on arrow-cue trials, 1.5 versus 1.3 letters, *t*(82) = 1.9, *p* = 0.053, *d* = 0.4. They reported more probes from the high- than from low-probability locations both on neutral-cue trials, 27% versus 18%, respectively, *t*(41) = 2.4, *p* = 0.009, *d* = 0.4 and on arrow-cue trials when the cued and high-probability locations coincided, 65% versus 9%, respectively, *t*(41) = 11, *p* < 0.001, *d* = 1.9. When the cued and high-probability locations did not coincide, participants reported more probes from the cued than from the high-probability location, 61% versus 13%, *t*(41) = 10, *p* < 0.001, and crucially, more from the high- than from low-probability locations, 13% versus 9%, respectively, *t*(41) = 1.9, *p* = 0.02, *d* = 0.3. The % of reported letters from the cued location tended to be larger when the cued and high-probability locations coincided than when did not, 65% versus 61%, respectively, *t*(41) = 1.26, *p* = 0.11, *d* = 0.2, mirroring the numerical trend on valid-cue search trials.

To summarize, during learning, LPL occurred on both search and probe trials, as in Experiment 1. The crucial finding concerned the extinction phase: on neutral-cue trials, even though participants knew that the location imbalance would be discontinued, search performance was better, and more letters were reported, for high- versus low-probability-target locations. Moreover, on arrow-cue trials, participants followed the explicit instruction to prioritize the pre-cued location and yet, the learnt bias was still evident. Specifically, during search, participants reoriented their attention faster to the high- than to low-probability locations on invalid-cue trials. On probe-report trials, most correctly reported letters were from the cued location, confirming that our cueing manipulation was effective; crucially, however, participants reported more letters from the (previously) high- versus low-probability locations.

Note that the bias on invalid-cue search trials might come from trials where participants did not use the cue (trials that would be equivalent to neutral-cue trials). A reasonable estimate of the proportion of such trials is the % of arrow-cue probe trials where participants reported the letter at the cued location (78.5%^3^). If participants were biased toward the high-probability location only on the remaining 21.5% of invalid-cue trials, they should show the same bias as on neutral-cue trials (127 ms). Accordingly, their overall bias should be 27 ms (78.5% × 0 ms + 21.5% × 127 ms)—much less than the reported 63-ms bias found on invalid-cue trials. Thus, this alternative account explains part but not all the bias on invalid-cue trials.

The bias on invalid-cue trials also reduces the likelihood that the proactive effect we reported might only reflect that participants moved their eyes to the high-probability location before probe-display onset. This alternative account predicts no advantage of the high-probability location on invalid-cue trials because it predicts that participants would move their eyes to the cued location and should not attend to the high-probability location thereafter.

Taken together, these findings show that attention is proactively and inflexibly allocated to the high-probability location and invalidate the possibility that the bias reflected voluntary, goal-directed attention.

## General discussion

The present findings provide a clear answer to our research question. We found that LPL established during one task transferred to another task involving different stimuli, a different goal and requiring a different response. We thus conclude that statistical learning of target location guides attention proactively (for a similar conclusion regarding distractor-location statistical learning, see Geng, 2014; Theeuwes et al., 2022; but see Chang et al., 2023).

Our experiments resolve the discrepancies between previous studies. By measuring the distribution of attention before it could be disengaged from the high-probability location, we increased the chances to observe LPL transfer, relative to Addleman et al.’s (2019) study. By probing the bias during extinction, we refute the intertrial-priming account that challenged Huang et al.’s (2022) conclusion. Moreover, we show that LPL generates a largely inflexible and long-lasting bias: Revealing to participants that the bias was no longer useful and diverting their attention to an arrow-cued location did not prevent them from prioritizing the high-probability location long after learning ended (see also Golan & Lamy, 2023).

Our findings clearly show that LPL induces proactive attentional shifts. It remains possible, however, that LPL might be both proactive and dynamically adaptable to context changes. This possibility is consistent with Jiang et al.’s (2015) finding that LPL established with one task did not transfer to a subsequent extinction phase where participants knew the task was different (as noted by Addleman et al., 2019). In our study, task changes were unpredictable, but the search task was more frequent than the probe task, and participants may therefore have prepared for the task context to remain constant on most trials. Further research is needed to determine whether cross-task transfer of LPL depends on whether or not participants know what task will come next.

## Supplementary Information

Below is the link to the electronic supplementary material.

Supplementary file1 (DOCX 12 KB)

Supplementary file2 (DOCX 32 KB)

## Footnotes

1 We used a difficult target-distractor discrimination, because a pilot study with an easier search task yielded a very small LPL effect.

2 In the Supplementary Materials, we provide additional analyses, with epoch (1–3) as a within-subject factor for the learning and extinction phases for Experiments 1 and 2.

3 Note that the % of *trials* where participants correctly reported the letter at the cued location (78.5%) is not the same as the % of *correctly reported probes* from the cued location (as explained in the Methods).

## Declarations

### Open practices statement

Data or materials for the experiments are available upon request, and none of the experiments was preregistered.
