# Supplementary material for: Statistical learning of target location guides attention proactively
Source: Psychon Bull Rev. 2025 May 21;32(5):2410–9. doi: 10.3758/s13423-025-02710-9 (PMC12425840; doi:10.3758/s13423-025-02710-9)
Supplement: Supplementary file 1 — Supplementary file1 (DOCX 12 KB) [file 13423_2025_2710_MOESM1_ESM.docx]

**Declarations**

**Funding**

The research is funded by the Israel Science Foundation (ISF) grants no 2449/21 to Dominique Lamy. The funders have/had no role in study design, data collection and analysis, decision to publish or preparation of the manuscript.

**Conflicts of interest/Competing interests**

The authors have no competing interests to declare.

**Ethics approval**

The research was approved by Tel Aviv University Ethics Committee, 0000285-5 and complies with all national and international (e.g., Declaration of Helsinki) ethical regulations.

**Consent to participate**

Informed consent was obtained from all participants.

**Consent for publication**

Informed consent was obtained from all participants.

**Availability of data and materials**

The data or materials for the experiments reported here will be made available upon request.

**Code availability**

The code will be made available upon request.

**Author’s contributions**

Aidai Golan and Dominique Lamy contributed to the conceptualization, methodology, and writing of this research. Aidai Golan collected the data and conducted the analyses. Aniruddha Ramgir programmed the Experiments and conducted the power analysis. Funding and supervision of this research was managed by Dominique Lamy.
