# Supplementary material for: Statistical learning of target location guides attention proactively
Source: Psychon Bull Rev. 2025 May 21;32(5):2410–9. doi: 10.3758/s13423-025-02710-9 (PMC12425840; doi:10.3758/s13423-025-02710-9)
Supplement: Supplementary file 2 — Supplementary file2 (DOCX 32 KB) [file 13423_2025_2710_MOESM2_ESM.docx]

**Supplementary Materials**

We added epoch (1 to 3) as a within-subject factor in the analyses reported in the main document.

**Experiment 1**

*Learning phase*

The mean RT, accuracy, and percentage of correct probe reports for each design cell is presented in Table 1. To avoid redundancy with the main text, we only report interactions involving epoch.

*Search trials*. We conducted an Analysis of Variance (ANOVA) with target-location probability (high vs. low probability) and epoch (1 to 3) as within-subject factors. On RTs, the interaction between target-location probability and epoch was significant, *F*(2,62)=10.38, p<.001, $\eta_{p}^{2}$=.25, indicating that the RT advantage on high- vs. low-probability location trials increased as the learning phase progressed. On accuracy, the interaction was not significant, *F*(2, 62)=1.08, p=.345, $\eta_{p}^{2}$=.03.

*Probe trials*. We conducted an ANOVA with reported-probe location (high vs. low probability) and epoch (1 to 3) as within-subject factors The interaction between epoch and reported-probe location was significant, *F*(2,62)=3.46, p=.05, $\eta_{p}^{2}$=.10, indicating that participants reported more probes from the high- than from the low-probability locations as the learning phase progressed.

**Table 1**: Mean reaction times (RTs, in milliseconds) and accuracy (in percentages) as a function of target-location probability (high vs. low) and mean correct probe reports (in percentages) as a function of reported-probe location (high vs. low probability), for each epoch (1 to 3) in the learning phase of Experiment 1. The numbers in brackets denote within-subject standard errors (Morey, 2008).

|  | Epoch 1 | | Epoch 2 | | Epoch 3 | |
| --- | --- | --- | --- | --- | --- | --- |
|  | High | Low | High | Low | High | Low |
| *Search RTs* | 869 [22] | 1129 [25] | 832 [20] | 1131 [24] | 788 [23] | 1139 [21] |
| *Search Accuracy* | 88% [2%] | 74% [2%] | 91% [1%] | 75% [1%] | 91% [1%] | 79% [1%] |
| *Probe Reports* | 35% [4%] | 16% [3%] | 38% [3%] | 15% [3%] | 41% [3%] | 15% [3%] |

*Extinction phase*

The mean RT, accuracy, and probe report rate for each design cell is presented in Table 2.

*Search trials*. We conducted an ANOVA with target-location probability (high vs. low) and epoch (1 to 3) as within-subject factors The interaction between target-location probability and epoch was not significant on either RTs, *F*(2, 62)=2.07, p=.13, $\eta_{p}^{2}$=.06 or accuracy, *F*<1,indicating that the learnt bias did not decrease during extinction.

*Probe trials*. We conducted an ANOVA with reported-probe location (high vs. low probability) and epoch (1 to 3) as within-subject factors The interaction between these factors was not significant, *F*<1.

**Table 2**: Mean reaction times (RTs, in milliseconds) and accuracy (in percentages) as a function of target-location probability (high vs. low), and mean correct probe reports (in percentages) as a function of reported-probe location (high vs. low probability), for each epoch (1 to 3) in the extinction phase of Experiment 1. The numbers in brackets denote within-subject standard errors (Morey, 2008).

|  | Epoch 1 | | Epoch 2 | | Epoch 3 | |
| --- | --- | --- | --- | --- | --- | --- |
|  | High | Low | High | Low | High | Low |
| *Search RTs* | 937 [21] | 1077 [22] | 951 [20] | 1072 [22] | 961 [30] | 1054 [17] |
| *Search Accuracy* | 88% [2%] | 83% [1%] | 87% [2%] | 83% [1%] | 87% [2%] | 84% [2%] |
| *Probe Reports* | 30% [3] | 18% [3] | 30% [3] | 17% [3] | 29% [3] | 18% [3] |

**Experiment 2**

*Learning phase*

The mean RT, accuracy, and probe-report rate for each design cell is presented in Table 3.

*Search trials*. We conducted an ANOVA with target-location probability (high vs. low) and epoch (1 to 3) and as within-subject factors. The interaction between target-location probability and epoch was significant, *F*(2,82)=6.0, p=.003, $\eta_{p}^{2}$=.13, indicating that the RT advantage on high- vs. low-probability location trials increased as the learning phase progressed. On accuracy, the interaction was not significant, *F*(2, 82)=1.98, p=.144, $\eta_{p}^{2}$=.05.

*Probe trials*. We conducted an ANOVA with reported-probe location (high vs. low probability) and epoch (1 to 3) and as within-subject factors. The interaction between reported-probe location and epoch was significant *F*(2,82)=7.18, p=.002, $\eta_{p}^{2}$=.15, indicating that participants reported more probes from the high- relative to low-probability locations as the learning phase progressed.

**Table 3**: Mean reaction times (RTs, in milliseconds), accuracy (in percentages) as a function of target-location probability (high vs. low) and mean correct probe reports (in percentages) as a function of reported-probe location (high vs. low probability), for each of epoch (1 to 3) in Experiment 2. The numbers in brackets denote within-subject standard errors (Morey, 2008).

|  | Epoch 1 | | Epoch 2 | | Epoch 3 | |
| --- | --- | --- | --- | --- | --- | --- |
|  | High | Low | High | Low | High | Low |
| *Search RTs* | 862 [21] | 1126 [19] | 797 [13] | 1124 [17] | 766 [14] | 1110 [17] |
| *Search Accuracy* | 84% [2%] | 70% [1%] | 89% [1%] | 74% [2%] | 91% [1%] | 71% [2%] |
| *Probe Reports* | 36% [3%] | 16% [2%] | 42% [2%] | 14% [2%] | 44% [3%] | 14% [2%] |

*Extinction phase*

*Search trials*. Experiment 2 was not powered to include epoch as a factor in the ANOVA, in addition to cue validity and target-location probability. As a result, some participants had empty cells in the valid-cue condition. Thus, we present the RTs and accuracy data only for invalid- and neutral-cue trials.

We conducted an ANOVA with epoch (1 to 3) and target location probability (high vs. low probability) as within-subject factors separately for invalid- and neutral-cue trials. The mean RT and accuracy rate for each design cell are presented in Table 4.

The interaction between target-location probability and epoch was not significant for either invalid- or neutral-cue trials, both on RTs, *F*<1 and *F*(2, 82) = 1.69, p = .191, $\eta_{p}^{2}$=.044, and on accuracy, both *Fs*<1.

**Table 4**: Mean reaction times (RTs, in milliseconds) and accuracy (in percentages), as a function of target-location probability (high vs. low) and epoch (1 to 3) for invalid- and neutral-cue trials in the extinction phase of Experiment 2. The numbers in brackets denote within-subject standard errors (Morey, 2008).

|  | Epoch 1 | | Epoch 2 | | Epoch 3 | |
| --- | --- | --- | --- | --- | --- | --- |
| Cue validity | High | Low | High | Low | High | Low |
| *RTs* |  |  |  |  |  |  |
| Invalid | 1188 [29] | 1258 [14] | 1163 [23] | 1219 [17] | 1094 [26] | 1182 [17] |
| Neutral | 1014 [21] | 1128 [21] | 969 [20] | 1120 [16] | 995 [23] | 1089 [17] |
| *Accuracy* |  |  |  |  |  |  |
| Invalid | 72% [3%] | 68% [2%] | 76% [3%] | 70% [2%] | 77% [2%] | 74% [1%] |
| Neutral | 83% [3%] | 76% [1%] | 86% [2%] | 76% [1%] | 83% [2%] | 77% [1%] |

*Probe trials*. We conducted an ANOVA with reported-probe location (high vs. low probability) and epoch (1 to 3) as within-subject factors separately for three cue-condition trials: (a) neutral-cue trials, (b) arrow-cue trials on which the cue location coincided with the high-probability location, and (c) arrow-cue trials in which the cue location coincided with a low-probability location. The mean percentage of correct probe reports for each design cell is presented in Table 5.

On neutral-cue trials, the interaction between reported-probe location and epoch was not significant, *F*<1, indicating that the bias remained constant during extinction. On arrow-cue trials for which the cue and high-probability locations coincided, the interaction between reported-probe location and epoch was significant, *F*(2,82)=7.73, p<.001, $\eta_{p}^{2}$=.16. Follow-up analyses revealed that the difference between cued high- and uncued low-probability locations was similar during the first two epochs, *F*(1, 41) = 1.25, p=.271, $\eta_{p}^{2}$=.03 and decreased from the second to the third epochs, *F*(1, 41)=9.34, p=.004, $\eta_{p}^{2}$=.18. Unsurprisingly, it remained nevertheless highly significant during the third epoch, *F*(1, 41)=63.24, p<.001, $\eta_{p}^{2}$=.61. On arrow-cue trials in which the cue location coincided with a low-probability location, the relevant comparison for the present purposes is between the uncued high- and uncued low-probability locations. We therefore conducted the analysis while excluding the cued-location condition. The interaction between reported-probe location and epoch was not significant, F(2, 82)=1.55, p=.217, $\eta_{p}^{2}$=.03.

**Table 5**: Mean percentage of correct probe reports as a function of the relevant reported-probe location conditions (cued, high probability, low probability) and epoch (1-3) for each cue condition (neutral, arrow-cue at the high-probability location, arrow-cue at a low-probability location) in the extinction phase of Experiment 2. The numbers in brackets denote within-subject standard errors (Morey, 2008).

|  | Neutral | |  | Cue at the high-prob. location | |  | Cue at a low-prob. location | | |
| --- | --- | --- | --- | --- | --- | --- | --- | --- | --- |
| Epoch | High | Low |  | High | Low |  | Cued - LP | Uncued - HP | Uncued LP |
| 1 | 27% [2%] | 18% [2%] |  | 72% [4%] | 7% [2%] |  | 65% [3%] | 11% [2%] | 8% [2%] |
| 2 | 28% [2%] | 18% [2%] |  | 69% [4%] | 8% [2%] |  | 60% [3%] | 12% [2%] | 9% [2%] |
| 3 | 27% [2%] | 18% [2%] |  | 58% [4%] | 10% [2%] |  | 61% [4%] | 14% [2%] | 8% [2%] |
